# Barbed Suture and Gastrointestinal Surgery. A Retrospective Analysis

**DOI:** 10.1515/med-2019-0055

**Published:** 2019-08-09

**Authors:** Michele Manigrasso, Nunzio Velotti, Federica Calculli, Giovanni Aprea, Katia Di Lauro, Enrico Araimo, Ugo Elmore, Sara Vertaldi, Pietro Anoldo, Mario Musella, Marco Milone, Loredana Maria Sosa Fernandez, Francesco Milone, Giovanni Domenico De Palma

**Affiliations:** 1 Department of Clinical Medicine and Surgery. University “Federico II” of Naples, Via S. Pansini 5, 80131 Naples, Italy; 2 Department of Surgical Sciences, University of Campania “Luigi Vanvitelli”, Via S. Pansini 5, 80131 Naples, Italy; 3 Department of Gastrointestinal Surgery, San Raffaele Scientific Institute, Via Olgettina 60, 20132, Milan, Italy

**Keywords:** Minimally invasive surgery, Laparoscopy, Intracorporeal anastomosis, Barbed suture

## Abstract

Although minimally invasive surgery is recognized as the gold standard of many surgical procedures, laparoscopic suturing is still considered as the most difficult skill in laparoscopic surgery.

The introduction of barbed sutures facilitates laparoscopic suturing because it is not necessary to tie a knot. The efficacy of this method has been evaluated in different types of surgery; however, less is known about general surgery.

We retrospectively analysed data from 378 patients who had undergone bariatric or surgical treatment for colic or gastric malignancy requiring a closure of gastroentero, entero-entero or enterocolotomy from January 2014 to January 2019, admitted to the General Surgery Unit and Operative Unit of Surgical Endoscopy of the University Federico II (Naples, Italy).

We registered 12 anastomotic leaks (3.1%), 16 anastomotic intraluminal bleedings (4.2%) and 7 extraluminal bleedings. Other complications included 23 cases of postoperative nausea and vomit (6%), 14 cases of postoperative ileus (3.7%) and 3 cases of intra-abdominal abscess (0.8%). Overall complications rate was 19.8% (75/378). No postoperative death was registered.

Thus, by pooling together 378 patients, we can assess that barbed suture could be considered safe and effective for closure of holes used for the introduction of a branch of mechanical stapler to perform intracorporeal anastomosis.

## 1 Introduction

Although minimally invasive surgery is nowadays considered as the gold standard of many surgical procedures [1, 2, 3, 4, 5, 6], laparoscopic suturing is still considered as the most difficult skill in laparoscopic surgery. The reason of this difficulty lies in the need to tie a knot in a little space, often with limited visualization.

The integrity of a knot is particularly important in the closure of anastomosis, because closure failure could cause serious complications, i.e. the need for a reintervention, prolonged hospital stay, and increased health care costs [7].

The introduction of barbed sutures facilitates laparoscopic suturing, because it is not necessary to tie a knot. The efficacy of this new method has been evaluated in different types of surgery [8, 9, 10, 11, 12, 13]; however, less is known about general surgery.

The aim of this study is to assess the efficacy and safety of barbed suture after different kind of anastomosis in general surgery.

## 2 Materials and methods

### 2.1 Study population

We retrospectively analyzed data from a prospectively maintained database of 378 patients who had undergone different surgical procedures requiring a closure of gastroentero- entero-entero or enterocolotomy. Patients who had undergone bariatric or surgical treatment for colic or gastric malignancy from January 2014 to January 2019, admitted to the General Surgery Unit and Operative Unit of Surgical Endoscopy of the Federico II University (Naples, Italy), were consecutively enrolled in this review. This study was approved by our institutional review board and the informed consent was obtained from all patients before enrolment. All investigations complied with the principles of the Declaration of Helsinki. All patients included in the study had undergone intracorporeal anastomosis in case of segmental colectomy or subtotal gastrectomy with Roux-en-Y anastomosis for malignancy and in case of bariatric surgery (Roux-en-Y gastric bypass, RYGB, or One-Anastomosis Gastric Bypass, OAGB/MGB).

### 2.2 Outcomes

All data and outcomes were recorded during the hospital stay.

Outcomes were divided into primary and secondary. Primary outcome was the rate of anastomotic leakage, defined as all conditions with clinical or radiological anastomotic dehiscence, with or without the need of surgical revision.

Secondary outcomes were represented by anastomotic bleeding, defined as any bleeding requiring blood transfusions, and the rate of other minor and major complications, defined as any deviation from the normal post operative course. Clavien-Dindo Classification was used to define minor (Clavien-Dindo 1-2) and major complications (Clavien-Dindo 3-4-5). Demographic data (age, sex, BMI, ASA Score) and the type of disease were also recorded.

### 2.3 Operative technique

According to the current literature peri-operative prophylaxis routinely included subcutaneous heparin administration and perioperative handling of anti-platelet drugs [14,15].

All bariatric and oncology procedures were performed by expert surgeons in laparoscopic surgery. In case of bariatric intervention, both gastroentero- and entero-enterotomy (in case of RYGB) were closed by deep corner suture with braided suture, and double layer running barbed suture.

In case of oncologic procedures, all enterotomies performed were closed by deep corner suture with non-braided suture and double layer running barbed suture.

Type of used suture and the use of double layer closure were related to surgeons’ habits.

## 3 Results

During the study period, we collected data of 378 patients, 210 males (55.5%) and 168 females (44.5%). Of them, 125 patients had undergone segmental colic or gastric resection for malignancy (32 splenic flexure resections, 73 right hemicolectomies and 20 subtotal gastrectomies), and 253

bariatric surgery for morbid obesity (210 MGB/OAGB and 43 RYGB). Demographic data of the group are summarized in Table 1. Mean age was 42.6±8.26, mean BMI of the whole group was 43.5±9.3 (27±4.3 in oncologic group and 47.2±5.2 in bariatric group). Mean ASA Score was 2.9±0.7.

**Table 1:** Patients’ and diseases’ characteristics.

Patients (N)	378
Gender (M)	210 (55.5%)
Patients (N)	378
Gender (M)	210 (55.5%)
Gender (F) Intervention	168 (44.5%)
Malignancy	125 (33%)
*Splenic flexure resection*	32
*Right hemicolectomy*	73
*Subtotal gastrectomy with Roux-en-Y reconstruction*	20
Morbid obesity	253 (67%)
*OAGB/MGB*	210
*RYGB*	43
Age (years)	42.6±8.26
Overall BMI	43.5±9.3
*BMI of patients with malignancy*	27±4.3
*BMI of obese patients*	47.2±5.2
ASA score	2.9±0.7

Primary and secondary outcomes are summarized in Table 2. Anastomotic leakage was detected in 12 patients (3.1%). Of these leaks, 4 were treated conservatively with antibiotics, 5 needed a radiological intervention and 3 required a surgical reintervention.

**Table 2:** Postoperative complications

Complications	75 (19.8%)
*Anastomotic leakage*	12 (3.1%)
*Intraluminal bleeding*	16 (4.2%)
*Extraluminal bleeding*	7 (1.9%)
*Nausea and vomit*	23 (6%)
*Postoperative ileus*	14 (3.7%)
*Intra-abdominal abscess*	3 (0.8%)

Anastomotic intraluminal bleeding was detected in 16 patients (4.2%). All the bleedings required transfusion and in 3 cases an endoscopic haemostasis was needed. Extraluminal bleeding was detected in 7 patients (1.9%) requiring blood transfusion. No bleedings required surgical re-intervention was reported.

Other complications included 23 cases of postoperative nausea and vomit (6%), 14 cases of postoperative ileus (3.7%) and 3 cases of intra-abdominal abscess (0.8%), with 1 requiring a CT-guided percutaneous drainage. Overall complications rate was 19.8% (75/378). No postoperative death was registered.

## 4 Discussion

Since its introduction in 1990s, minimally invasive surgery has gained widespread acceptance in many surgical fields due to its safety and advantages that this kind of procedure can offer as compared to open surgery [16, 17, 18, 19, 20, 21, 22, 23].

In fact, laparoscopic approach is associated with earlier recovery, less pain and improved cosmetic results.

Furthermore, the introduction of intracorporeal anastomosis and the possibility to perform a totally laparoscopic intervention has increased these differences with open surgery, even if the real benefits of intracorporeal

approach in certain fields of gastrointestinal surgery should be definitively assessed [24, 25, 26, 27, 28, 29, 30, 31, 32, 33, 34, 35].

Nevertheless, the possibility to perform intracorporeal anastomosis in minimally invasive surgery has underlined the importance of being able to perform a laparoscopic knot, the most difficult skill in laparoscopic surgery. In fact, gastroentero-, enteroentero- and coloenterotomy closure in minimally invasive procedures have always been challenging and related with serious complications (anastomotic leakage, intraabdominal abscess and stenosis).

With the introduction of barbed sutures, the challenge of laparoscopic suturing has been made easier.

The principal advantage of barbed sutures is the presence of barbs to anchor the suture to tissues in a knotless fashion, avoiding the need to tie a knot in a confined space.

In the current literature, the safety and efficacy of barbed suture has been demonstrated in many surgical fields [8, 9, 10, 11, 12, 13], but less is known in digestive surgery, where its efficacy and safety has been demonstrated mainly in bariatric surgery [36, 37, 38].

Recently, several studies are focused on barbed sutures to close gastrointestinal anastomosis after gastrectomy for cancer [39, 40, 41].

Bautista *et al*. [39], when performing barbed suture after RYGB, or Billroth II anastomosis after subtotal gastrectomies for malignancy in 50 patients, encountered one anastomotic leakage and no bleedings or stenosis. The authors concluded that intracorporeal enterotomy closure with barbed sutures is safe and effective.

In 2018, Lee SW [41] described a modified crossover technique for intracorporeal esophagojejunostomy following laparoscopic total gastrectomy involving 27 patients.

With this technique, Lee SW obtained no anastomotic leakage or other anastomosis-related complications, concluding that this technique could be considered safe and effective and would promote laparoscopic total gastrectomy as a promising surgical option.

Regarding enterotomy closure, some authors assessed a safety and efficacy of barbed suture [42,43].

In the case-control study of a prospectively maintained database of 94 patients (47 underwent barbed suture for enterotomy closure, and 47 conventional closure of enterotomy after right hemicolectomy with intracorporeal anastomosis), Feroci *et al*. reported 1 anastomotic leakage per group (2.12%) [42]. The authors concluded that the use of barbed suture could be considered as a safe and effective procedure for enterotomy closure.

A most recent study of a multi-centre retrospective case-control 80 patients analysis, after right colectomy with intracorporeal anastomosis, was published in 2018 by Bracale *et al*. [43].

While analysing data obtained from patients included in the study and divided into 2 groups (40 underwent enterotomy closure with barbed suture, and 40 conventional enterotomy closure), 2 anastomotic leaks, one in each group, were registered. Based on the data obtained from this study, the authors concluded that the use of a barbed suture for enterotomy closure can be considered safe and effective for completion of the stapled anastomosis in performing totally laparoscopic right colectomy.

The results obtained from our retrospective analysis are very encouraging and they are in line with the current literature.

In fact, in our analysis on 378 patients we registered 12 anastomotic leaks (3.1%) with only 3 requiring surgical reintervention (Clavien 3B). Anastomotic intraluminal bleeding was detected in 16 patients (4.2%). All bleedings required transfusion and in 3 cases an endoscopic haemostasis was needed (Clavien 3A). Extraluminal bleeding was detected in 7 patients (1.9%), requiring blood transfusion. No bleeding required surgical reintervention was registered. Other complications included 23 cases of postoperative nausea and vomit (6%) (Clavien 1), 14 cases of postoperative ileus (3.7%) (Clavien 1), and 3 cases of intra-abdominal abscess (0.8%) with a CT-guided percutaneous drainage required in 1 case (Clavien 3A). Overall complications rate was 19.8% (75/378). No postoperative death was registered.

The complication rate is even lower considering that RYGB and subtotal gastrectomy for malignancy required two anastomosis.

Thus, by pooling together 378 patients, we can assess that barbed suture could be considered safe and effective for a closure of the holes used for the introduction of the branch of mechanical stapler to perform anastomosis.

However, some limitations of this study have to be addressed. The most important limitation is the study design: this is in fact a retrospective analysis, with the lack of a comparison between groups and no randomization. Moreover, this is a monocentric study, in which always the same surgeons performed the procedures.

In conclusions, even if we can consider the use barbed suture safe and effective, further comparative studies are needed to give definitive conclusions.

## References

[1] Tinmouth J, Tomlinson G (2004). Laparoscopically assisted versus open colectomy for colon cancer. N Engl J Med.

[2] Guillou P, Quirke P, Thorpe H et al Short-term endpoints of conventional versus laparoscopic-assisted surgery in patients with colorectal cancer (MRC CLASICC trial): multicentre randomized controlled trial. Lancet.

[3] Lacy AM, Garcia-Valdecasas JC, Pique JM (1995). Short term outcome analysis of a randomized study comparing laparoscopic vs open colectomy for colon cancer. Surg Endosc.

[4] Milone M, Manigrasso M, Burati M, Velotti N, Milone F, De Palma GD (2018). Surgical resection for rectal cancer. Is laparoscopic surgery as successful as open approach? A systematic review with meta-analysis. PLoS One.

[5] Bianchi PP, Rosati R, Bona S, Rottoli M, Elmore U, Ceriani C, Malesci A, Montorsi M (2007). Laparoscopic surgery in rectal cancer: a prospective analysis of patient survival and outcomes. Dis Colon Rectum.

[6] The COLOR Study Group. COLOR: a randomized clinical trial comparing laparoscopic and open resection for colon cancer. Dig Surg.

[7] Ritter EM, Mcclusky DA, Gallagher AG et al Real-time objective assessment of knot quality with a portable tensiometer is superior to execution time for assessment of laparoscopic knottying performance. Surg Innov.

[8] Greenberg JA (2010). The use of barbed sutures in obstetrics and gynecology. Rev Obstet Gynecol.

[9] Einarsson JI, Chavan NR, Suzuki Y Use of bidirectional barbed suture in laparoscopic myomectomy: evaluation of perioperative outcomes, safety, and efficacy. J Minim Invasive Gynecol.

[10] Mansour A, Ballard R, Garg S, Baulesh D, Erickson M The use of barbed sutures during scoliosis fusion wound closure: a quality improvement analysis. J Pediatr Orthop.

[11] Shah HN, Nayyar R, Rajamahanty S, Hemal A Prospective evaluation of unidirectional barbed suture for various indications in surgeon-controlled robotic reconstructive urologic surgery: Wake Forest university experience. Int Urol Nephrol.

[12] De Blacam C, Colakoglu S, Momoh A, Lin S, Tobias A, Lee B (2012). Early experience with barbed sutures for abdominal closure in deep inferior epigastric perforator flap breast reconstruction. ePlasty.

[13] Warner JP, Gutowski KA Abdominoplasty with progressive tension closure using a barbed suture technique. Aesthet Surg J.

[14] Samama CM (2008). [Perioperative venous thromboembolism prophylaxis: short review and recommendations]. Ann Fr Anesth Reanim.

[15] Di Minno MND, Milone M, Mastronardi P, Ambrosino A, Di Minno A, et al (2013). Perioperative handling of antiplatelet drugs. A critical appraisal. Current Drug Targets.

[16] Oxley SG, Mallick R, Odejinmi F (2019). Laparoscopic myomectomy: an alternative approach to tackling submucous fibroids?. J Minim Invasive Gynecol.

[17] Milone M, Vignali A, Milone F, Pignata G, Elmore U, Musella M, De Placido G, Mollo A, Fernandez LM, Coretti G, Bracale U, Rosati R (2015). Colorectal resection in deep pelvic endometriosis: Surgical technique and post-operative complications. World J Gastroenterol.

[18] Milone M, Fernandez LM, Musella M, Milone F (2016). Safety and Efficacy of Minimally Invasive Video-Assisted Ablation of Pilonidal Sinus: A Randomized Clinical Trial. JAMA Surg.

[19] (2018). 2017 and 2015 European Society of Coloproctology (ESCP) collaborating groups. The impact of conversion on the risk of major complication following laparoscopic colonic surgery: an international, multicentre prospective audit. Colorectal Dis.

[20] Milone M, Elmore U, Vignali A, Mellano A, Gennarelli N, Manigrasso M, Milone F, De Palma GD, Muratore A, Rosati R (2017). Pulmonary Complications after Surgery for Rectal Cancer in Elderly Patients: Evaluation of Laparoscopic versus Open Approach from a Multicenter Study on 477 Consecutive Cases. Gastroenterol Res Pract.

[21] Milone M, Elmore U, Musella M, Parise P, Zotti MC, Bracale U, Di Lauro K, Manigrasso M, Milone F, Rosati R. (2017). Safety and efficacy of laparoscopic wedge gastrectomy for large gastrointestinal stromal tumors. Eur J Surg Oncol.

[22] Conzo G, Musella M, Corcione F, De Palma M, Ferraro F, Palazzo A, Napolitano S, Milone M, Pasquali D, Sinisi AA, Colantuoni V, Santini L (2013). Laparoscopic adrenalectomy, a safe procedure for pheochromocytoma. A retrospective review of clinical series. Int J Surg.

[23] Martínez-Pérez A, Carra MC, Brunetti F, de’Angelis N (2017). Short-term clinical outcomes of laparoscopic vs open rectal excision for rectal cancer: A systematic review and meta-analysis. World J Gastroenterol.

[24] Feroci F, Lenzi E, Garzi A, Vannucchi A, Cantafio S, Scatizzi M (2013). Intracorporeal versus extracorporeal anastomosis after laparoscopic right hemicolectomy for cancer: a systematic review and meta-analysis. Int J Colorectal Dis.

[25] Milone M, Elmore U, Vignali A, Gennarelli N, Manigrasso M, Burati M, Milone F, De Palma GD, Delrio P, Rosati R (2018). Recovery after intracorporeal anastomosis in laparoscopic right hemicolectomy: a systematic review and meta-analysis. Langenbecks Arch Surg.

[26] Ricci C, Casadei R, Alagna V, Zani E, Taffurelli G, Pacilio CA, Minni F (2017). A critical and comprehensive systematic review and meta-analysis of studies comparing intracorporeal and extracorporeal anastomosis in laparoscopic right hemicolectomy. Langenbecks Arch Surg.

[27] Milone M, Elmore U, Di Salvo E, Delrio P, Bucci L, Ferulano GP, Napolitano C, Angiolini MR, Bracale U, Clemente M, D’ambra M, Luglio G, Musella M, Pace U, Rosati R, Milone F (2015). Intracorporeal versus extracorporeal anastomosis. Results from a multicentre comparative study on 512 right-sided colorectal cancers. Surg Endosc.

[28] Stein SA, Bergamaschi R (2013). Extracorporeal versus intracorporeal ileocolic anastomosis. Tech Coloproctol.

[29] Vignali A, Elmore U, Lemma M, Guarnieri G, Radaelli G, Rosati R (2018). Intracorporeal versus Extracorporeal Anastomoses Following Laparoscopic Right Colectomy in Obese Patients: A Case-Matched Study. Dig Surg.

[30] Milone M, Angelini P, Berardi G, Burati M, Corcione F, Delrio P, Elmore U, Lemma M, Manigrasso M, Mellano A (2018). Intracorporeal versus extracorporeal anastomosis after laparoscopic left colectomy for splenic flexure cancer: results from a multi-institutional audit on 181 consecutive patients. Surg Endosc.

[31] Grieco M, Cassini D, Spoletini D, Soligo E, Grattarola E, Baldazzi G, Testa S, Carlini M (2019). Intracorporeal Versus Extracorporeal Anastomosis for Laparoscopic Resection of the Splenic Flexure Colon Cancer: A Multicenter Propensity Score Analysis. Surg Laparosc Endosc Percutan Tech.

[32] Swaid F, Sroka G, Madi H, Shteinberg D, Somri M, Matter I (2016). Totally laparoscopic versus laparoscopic-assisted left colectomy for cancer: a retrospective review. Surg Endosc.

[33] Milone M, Manigrasso M, Burati M, Elmore U, Gennarelli N, Cesare Giglio M, Maione F, Musella M, Lo Conte V, Milone F, Domenico De Palma G (2019). Intracorporeal versus extracorporeal anastomosis after laparoscopic gastrectomy for gastric cancer. A systematic review with meta-analysis. J Visc Surg.

[34] Chen K, Pan Y, Cai JQ, Wu D, Yan JF, Chen DW, Yu HM, Wang XF (2016). Totally laparoscopic versus laparoscopic-assisted total gastrectomy for upper and middle gastric cancer: a single-unit experience of 253 cases with meta-analysis. World J Surg Oncol.

[35] Chen K, Mou YP, Xu XW, Pan Y, Zhou YC, Cai JQ, Huang CJ (2015). Comparison of short-term surgical outcomes between totally laparoscopic and laparoscopic-assisted distal gastrectomy for gastric cancer: a 10-y single-center experience with meta-analysis. J Surg Res.

[36] Lin Y, Long Y, Lai S, Zhang Y, Guo Q, Huang J, Du L (2019). The Effectiveness and Safety of Barbed Sutures in the Bariatric Surgery: a Systematic Review and Meta-analysis. Obes Surg.

[37] Pennestrì F, Gallucci P, Prioli F, Giustacchini P, Ciccoritti L, Sessa L, Bellantone R, Raffaelli M (2019). Barbed vs conventional sutures in bariatric surgery: a propensity score analysis from a high-volume center. Updates Surg.

[38] Milone M, Di Minno MN, Galloro G, Maietta P, Bianco P, Milone F, Musella M (2013). Safety and efficacy of barbed suture for gastrointestinal suture: a prospective and randomized study on obese patients undergoing gastric bypass. J Laparoendosc Adv Surg Tech A.

[39] Bautista T, Shabbir A, Rao J, So J, Kono K, Durai P (2016). Enterotomy closure using knotless and barbed suture in laparoscopic upper gastrointestinal surgeries. Surg Endosc.

[40] Lee SW, Kawai M, Tashiro K, Nomura E, Tokuhara T, Kawashima S, Tanaka R, Uchiyama K (2016). Laparoscopic gastrointestinal anastomoses using knotless barbed absorbable sutures are safe and reproducible: a single-center experience with 242 patients. Jpn J Clin Oncol.

[41] Lee SW, Kawai M, Tashiro K, Kawashima S, Tanaka R, Tanaka K, Nomura E, Uchiyama K (2018). The crossover technique for intracorporeal esophagojejunostomy following laparoscopic total gastrectomy: a simple and safe technique using a linear stapler and two barbed sutures. Surg Endosc.

[42] Feroci F, Giani I, Baraghini M, Romoli L, Zalla T, Quattromani R, Cantafio S, Scatizzi M (2018). Barbed versus traditional suture for enterotomy closure after laparoscopic right colectomy with intracorporeal mechanical anastomosis: a case-control study. Updates Surg.

[43] Bracale U, Merola G, Cabras F, Andreuccetti J, Corcione F, Pignata G (2018). The Use of Barbed Suture for Intracorporeal Mechanical Anastomosis During a Totally Laparoscopic Right Colectomy: Is It Safe? A Retrospective Nonrandomized Comparative Multicenter Study. Surg Innov.

